# Impact of Intron and Retransformation on Transgene Expression in Leaf and Fruit Tissues of Field-Grown Pear Trees

**DOI:** 10.3390/ijms241612883

**Published:** 2023-08-17

**Authors:** Vadim Lebedev

**Affiliations:** Branch of the Shemyakin-Ovchinnikov Institute of Bioorganic Chemistry of the Russian Academy of Sciences, 142290 Pushchino, Russia; vglebedev@mail.ru

**Keywords:** biosafety, expression stability, herbicide resistance, intron-mediated enhancement, *Pyrus communis*, transgene stacking, transgenic fruits, transgrafting, *uidA* gene, unintended effects

## Abstract

Stable and high expression of introduced genes is a prerequisite for using transgenic trees. Transgene stacking enables combining several valuable traits, but repeated transformation increases the risk of unintended effects. This work studied the stability and intron-mediated enhancement of *uidA* gene expression in leaves and different anatomical parts of pear fruits during field trials over 14 years. The stability of reporter and herbicide resistance transgenes in retransformed pear plants, as well as possible unintended effects using high-throughput phenotyping tools, were also investigated. The activity of β-glucuronidase (GUS) varied depending on the year, but silencing did not occur. The *uidA* gene was expressed to a maximum in seeds, slightly less in the peel and peduncles, and much less in the pulp of pear fruits. The intron in the *uidA* gene stably increased expression in leaves and fruits by approximately twofold. Retransformants with the *bar* gene showed long-term herbicide resistance and exhibited no consistent changes in leaf size and shape. The transgenic pear was used as rootstock and scion, but grafted plants showed no transport of the GUS protein through the graft in the greenhouse and field. This longest field trial of transgenic fruit trees demonstrates stable expression under varying environmental conditions, the expression-enhancing effect of intron and the absence of unintended effects in single- and double-transformed woody plants.

## 1. Introduction

The stability of transgenic traits throughout the entire plant life is very important for perennial woody plants. Unstable expression or silencing of transgenes is a key problem not only for their commercial use but also for biosafety, for example, when it comes to reproductive sterility traits [1]. Loss of expression may be a consequence of either epigenetic effects or the transformation process [2]. Initially, changes in expression can be induced by stresses such as changes in growth conditions (transfer from the greenhouse to the field) [3] or first overwintering [4,5]. In addition, the duration of tree growth can reach decades, and during this time, they go through numerous cycles of dormancy and growth and are exposed to extreme changes in various environmental conditions and the effects of biotic stresses. It has been repeatedly shown that gene silencing can occur under the influence of environmental stresses [6,7,8]. Environment-induced changes in transgene expression are complex and largely unpredictable, and their study requires field trials of transgenic trees over many years [4]. However, the number of long-term field trials of transgenic fruit trees is limited. The stability and distribution of transgene expression in parts of transgenic fruits have been little studied.

The introduction of multiple genes (transgene “stacking” or “pyramiding”) in crops allows the combining of several new characteristics in one plant or improving agronomic polygenic traits [9]. In 2019, stacked transgenic varieties of maize, soybean, and cotton with insect resistance and herbicide tolerance occupied almost half (45%) of the global biotech crop area [10]. These annual stacked crops are obtained by sexual crossing, but the stacking of transgenes in vegetatively propagated plants, including perennial fruit trees, is produced by sequential or repeated transformation [11]. However, the risk of expression instability associated with the transformation process increases for stacked transgenic plants obtained by retransformation. Research into the expression stability of retransformed transgenic trees is very limited. Furthermore, apart from changes or silencing of transgene expression, unintended effects that go beyond the primary expected effects of genetic modification can be induced in transgenic plants [12]. They are the result of disruption due to gene insertion, random mutations during the transformation and tissue culture process, or pleiotropic effects of the introduced protein, and there is no single direct test for them [13]. Very few studies on expression stability and unintended effects of retransformed transgenic trees are known. These problems have been widely discussed in other organisms, such as fungi [14].

The high level of transferred gene expression in plants is no less important than its stability. This is generally achieved using strong constitutive promoters, but other methods are also known, e.g., intron-mediated enhancement (IME). This phenomenon in plants was first demonstrated in maize cells in 1987 [15]. This technique has long been used in plant genetic engineering, and recently, IME has been successfully applied to improve the efficiency of genome editing [16]. This phenomenon has been long studied, but the mechanisms of its action are still not understood [17]. To the best of our knowledge, the IME of expression in trees has not yet been studied.

Grafting is an ancient horticultural practice that enables combining in one plant the properties of different organisms—rootstock and scion. It is used most often in fruit tree nurseries. Rootstocks are most commonly used for control of tree size and architecture, but they can also increase pest and disease tolerance, adaptability to abiotic stresses, accelerate flowering, increase yields, and improve fruit quality [18,19]. Communication between rootstock and scion occurs through the transport of phytohormones, DNA, RNA and proteins across graft union [20]. The use of transgrafting, when one of the two components in the rootstock–scion combination is genetically modified, can further enhance the advantages of this system due to the traits encoded by transgenes [21]. Moreover, a transgenic interstock can be used in a more complex three-component system. Li et al. [22] showed that interstock can influence various biological processes in both scions and rootstocks in apple trees. Transgenic rootstocks enhanced virus resistance of the non-transgenic sweet cherry scions [23], induced precocious flowering in sweet oranges [24], and enhanced tolerance of apple scions to long-term drought stress [25]. The use of only transgenic rootstock gives the following advantages: (1) the transgene flow through pollen, and the occurrence of a transgenic product in fruits are prevented; (2) the number of rootstocks is significantly less than that of scions, and one approved rootstock can be used with many cultivars; (3) traits not necessary for scion, for example, herbicide resistance for weed control in rootstock nurseries, can be transferred to the rootstock. However, the presence of mobile transgenic products in non-transgenic scions may be a food-safety issue, and the potential transport of DNA, RNA and proteins from the rootstock across the graft needs to be assessed [26]. Despite a number of studies, the transport of transgenic molecules through the graft is still uncertain and requires further research [27].

The aim of this study was to investigate the stability and intron-mediated enhancement of *uidA* gene expression in leaves and different anatomical parts of pear fruits during long-term field trials. The β-glucuronidase gene *uidA* (GUS) remains a favorite reporter gene in plant molecular biology due to its sensitivity, stability, versatility and independence from plant metabolism [28,29]. In addition, we evaluated the stability of the reporter *uidA* gene, herbicide resistance of the *bar* gene, and the unintended effects in retransformed pear plants using advanced methods of phenotypic analysis. Finally, the bidirectional movement of the GUS protein across the graft between pear rootstock and scion was analyzed.

## 2. Results

### 2.1. Transgene Expression in Leaves

First, we investigated the stability and IME in the leaves of pear trees. Transgenic pear trees showed no deviations in growth and development from the non-transgenic control during field tests. The expression of the *uidA* gene in leaves was determined quantitatively over 11 years, starting from the year after planting in the field (Table 1). Silencing of expression was not observed: the level of activity in transgenic lines was always several times higher than the endogenous activity of β-glucuronidase in the control, which averaged 0.04 pmol 4-MU/min/µg protein over the years of measurements. The expression level varied significantly between years, but a consistently high enzyme activity was observed in lines HA-1, HA-3, HA-4, HB-1, NII-1 (on average, 6.0–10.7 pmol 4-MU/min/µg protein) and consistently low in lines HA-2, NIII-5, HA-7, NI-1 (on average, 0.3–1.1 pmol 4-MU/min/µg protein). The other lines had an intermediate level of expression, on average from 1.5 to 5.5 pmol 4-MU/min/µg protein. No dependence of the expression level on the year was observed, except for 2010 (a decrease) and 2011 (an increase for most lines).

**Figure 1:**
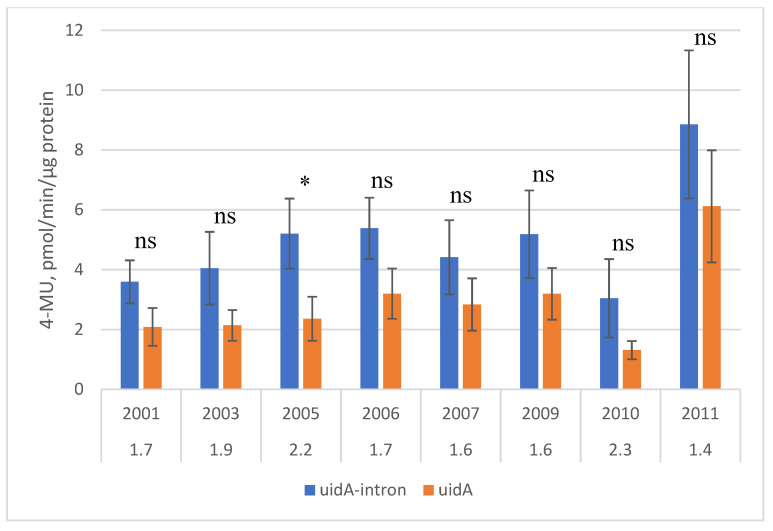
IME of *uidA* gene expression in leaves of transgenic pear. Average values for 10 transgenic lines with the *uidA*-intron gene and 9 with the *uidA* gene. The numbers under the years represent the degree of intron-mediated enhancement of *uidA* gene expression. Significance of differences measured by *t*-test (*: *p*-value < 0.05, ns—not significant).

Throughout the observation period, the average level of GUS activity was higher in the group of transgenic plants that expressed the *uidA* gene with an intron (10 lines) as compared with the group of plants containing the gene without an intron (9 lines). Despite significant fluctuations of expression in some lines, the IME of expression of the *uidA* gene in pear leaf tissue in the field was sufficiently stable and varied, depending on the year, from 1.4 to 2.3 times (Figure 1).

### 2.2. Transgene Expression in Fruits

**Figure 2:**
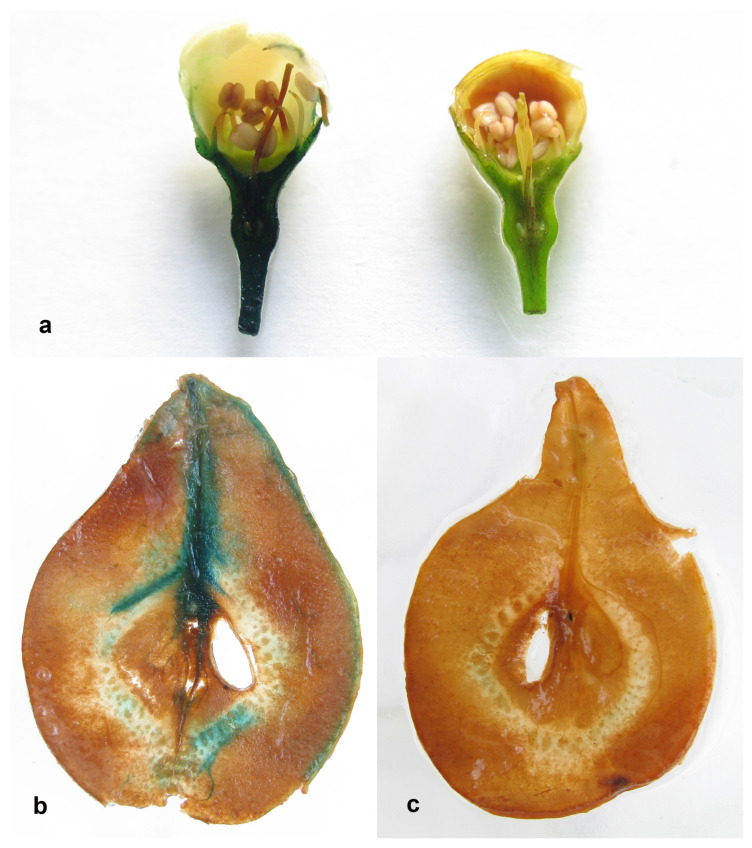
Histochemical GUS analysis of pear generative organs: (**a**) transgenic (left) and wild-type (right) flowers; (**b**) transgenic fruit; (**c**) wild-type fruit. GUS expression is shown as blue color in the flower and fruit of the transgenic pear plant.

**Figure 3:**
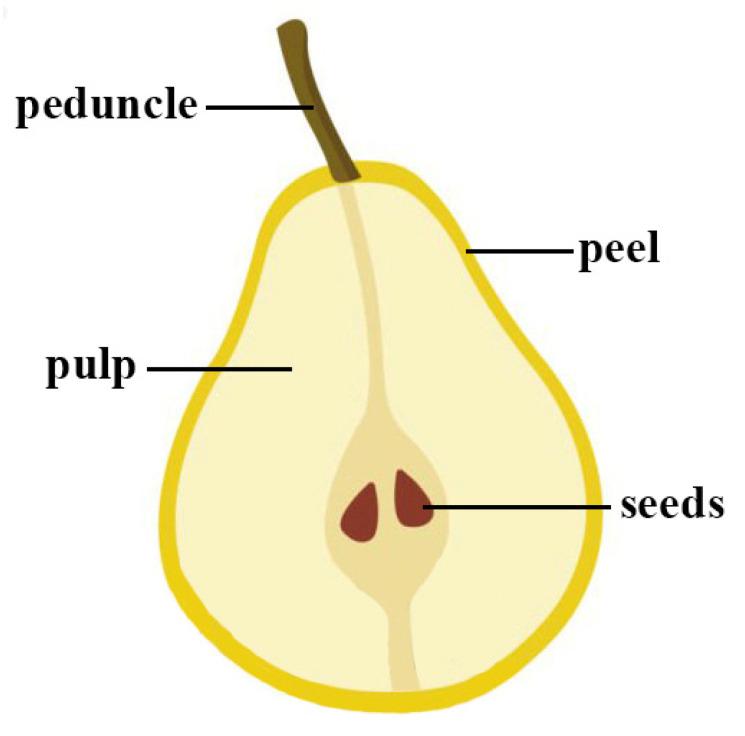
Pear fruit parts analyzed for *uidA* gene expression.

**Figure 4:**
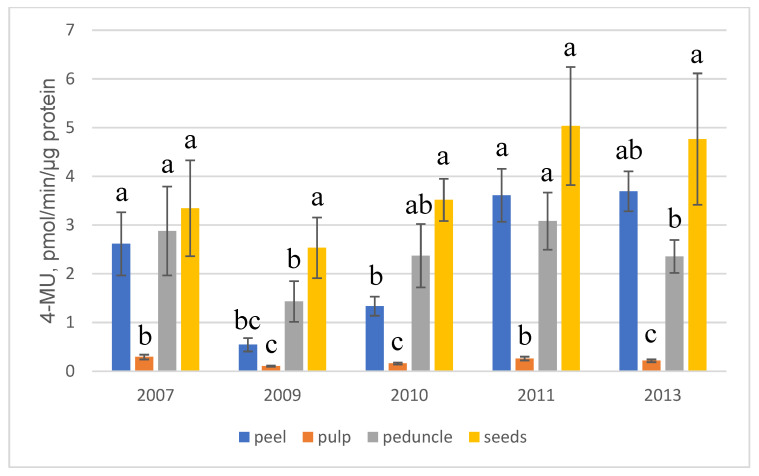
Levels of *uidA* gene expression in pear fruit tissues. Data are means ± SE (*n* = 6–11). Different letters indicate significant differences at *p* < 0.05.

Next, we examined the stability and IME in the pear fruits. Histochemical analysis showed the expression of the *uidA* gene in generative organs of the pear—the flower and fruit tissues (Figure 2). In fruits, the coloration was intense in the area of the peel and vascular tissue. Quantitative transgene expression analysis in pear fruits was carried out for 7 years; separate analyses for the peel, pulp, seeds and peduncle were conducted (Figure 3 and Figure 4).

Silencing of transgene expression in pear fruits was not observed, but the activity of β-glucuronidase in different parts of the fruit strongly varied. Over the entire observation period, it changed in the peel from 0.18 to 6.01 pmol 4-MU/min/µg protein; in the pulp, from 0.06 to 0.76; in the peduncle, from 0.31 to 8.30 and in the seeds from 0.65 to 16.20 pmol 4-MU/min/µg protein (Table S1). The expression level in the pulp was many times lower than in other parts of the fruit, but it was always higher than the endogenous activity of GUS in the pulp of non-transgenic fruits, which did not exceed 0.016 pmol 4-MU/min/µg protein. Among other parts, expression was maximal in seeds, which were slightly ahead of the peel and peduncle (Figure 4). In 2007, 2009 and 2010, expression was higher in the peduncle but in the peel in 2011 and 2013. Although the analysis of expression in leaves and fruits was carried out at different times (in July and August, respectively), expression in leaves was higher on the whole, except for a similar level in both organs in 2010. 

**Figure 5:**
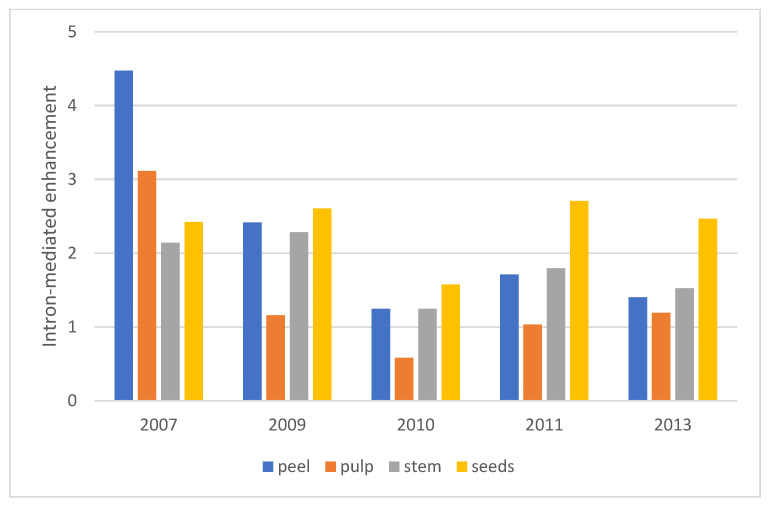
IME of *uidA* gene expression in transgenic pear fruits. Comparison of average values for 3–5 transgenic lines with the *uidA*-intron gene and 3–6 lines with the *uidA* gene.

IME of *uidA* gene expression was observed not only in pear leaves but also in fruits, which was seen from a comparison of the average level of enzyme activity in 3–5 lines with the gene and an intron and 3–6 lines with the gene without an intron (Figure 5). Enhancement was almost absent in the pulp (with the exception of 2007) but was present and was similar in the peel, peduncle and seeds. The level of IME in these parts of the fruit varied between years (from 1.2 to 4.5) but was twofold on average.

### 2.3. Transgene Expression in Retransformants

To evaluate the effect of repeat transformation on the stability of expression of the *uidA* gene and the appearance of unintended effects, we used transgenic plants obtained by retransformation with the herbicide resistance *bar* gene. Activity was measured for 10 years, starting from the year after planting in the field (Table 2). Retransformed lines demonstrated stability and no silencing but featured significant fluctuations in activity over the years. In addition, as shown by the color gradient, a separation into lines with high and low expression was observed among the lines obtained from the same initial genotype. For example, among the four lines obtained by transformation of line NII-1, there were two lines with high (T-AP, T-BT) and two lines with low (T-BO, T-CF) expression. Three lines originating from line NIV-2 also featured high (lines T2-BR and T2-BS) and low (line T2-DE) expression levels. These differences in expression levels were also stable. Transformation by the *bar* gene did not by itself cause a change in the level of β-glucuronidase endogenous activity (line P-BK) as compared with the non-transgenic control.

**Figure 6:**
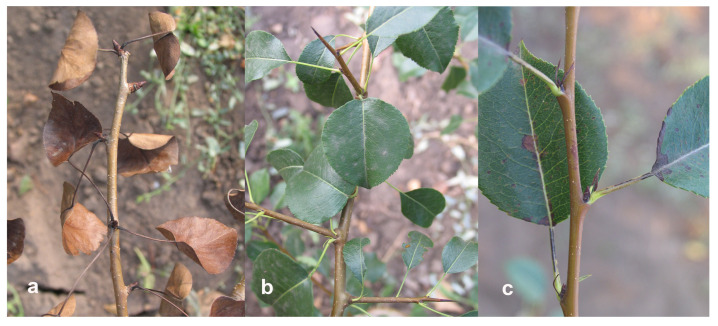
Herbicide damage after Basta application of pear branches: (**a**) non-transgenic control; (**b**) typical high-resistant line with the *bar* gene; (**c**) line T-CF.

Herbicide resistance of pear plants with the *bar* gene was assessed in the 10th year of field trials. Branches of 17 transgenic lines and of the control were treated with a 1% solution of Basta herbicide at a dose equivalent to 10 L/ha (a double field dose). After 3 days, complete leaf necrosis occurred in the non-transgenic control, whereas 16 transgenic lines showed no signs of damage (Figure 6a,b). Damage was observed only in line T-CF: some leaves had partial necrosis of leaf blades, petioles and stipules (Figure 6c). After 7 days, the leaves of the control plants fell off, but no changes occurred in the transgenic plants.

### 2.4. Identification of Potential Unintended Effects

To detect possible unintended changes in the phenotype of transgenic pear trees, we assessed the qualitative traits visually and the quantitative traits of the leaves using phenomics tools. We compared 33 phenotypical traits of a whole tree, shoots, leaves, flowers, fruits and seeds from the Guidelines for the conduct of tests for distinctness, uniformity and stability, which is used for testing new pear cultivars (Table S2). All transgenic pear lines showed no deviations of growth and development from the wild-type plants over three years. 

Though the transgenic pear trees showed no visible changes relative to the non-transgenic control, the visual assessment is subjective and is better to be confirmed by quantitative analysis. To identify possible unintended effects, the precision phenomic LAMINA software was used to measure pear leaf size and shape traits for three consecutive seasons (Figure S1). The first year of measurements found no significant differences between the leaves of transgenic lines with the *uidA* gene and the control (Figure S2). In the next two years, some transgenic lines demonstrated significant deviations in a number of parameters, but they were inconsistent and did not repeat. Similar results were obtained for transgenic lines with the *bar* gene (Figure S3). Significant deviations from the control were observed in all three years, but they were rare and did not repeat in any line for all three years. The frequency of these deviations was similar in pear transformants and retransformants.

### 2.5. Protein Transport across Draft

**Figure 7:**
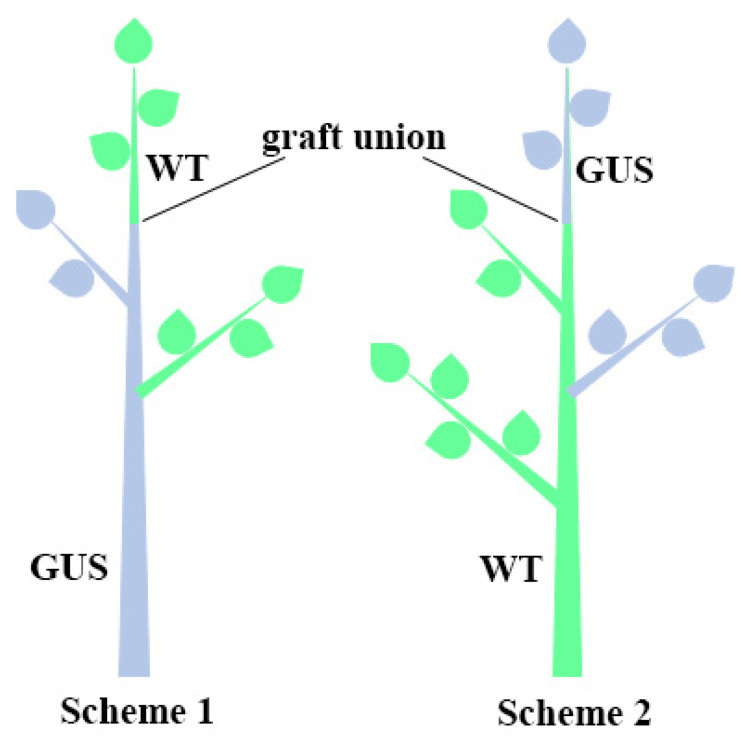
Schematic illustration of transgrafting experiment in pear. WT—wild-type component (green color), GUS—transgenic component (blue color).

To assess the transport of GUS protein through the graft in both directions (from rootstock to scion and from scion to rootstock), as well as into lateral branches, two graftings were made for each rootstock. One of them was left as the tip; the other was made a lateral branch (Figure 7). Additionally, lateral branches were left in the rootstock for expression control. A total of 10 plants were obtained: 4 for line HB-4 with the *uidA*-intron gene (2 per each Schemes 1 and 2) and 6 for line NII-1 with the *uidA* gene without an intron (3 per each Schemes 1 and 2). The transport was assessed for two years in the greenhouse (in the year of grafting and the subsequent year) and two years in the field (the next years after planting) by quantitative analyzing the GUS activity in leaf tissue. The *uidA* gene was stably expressed in transgenic rootstocks and scions for 10 years (Table 3). The transfer from the greenhouse to the field did not significantly affect the level of expression in transgenic tissue. However, in the leaves of non-transgenic branches, both grafted on transgenic plants and being a rootstock for transgenic lines, the level of β-glucuronidase activity did not exceed the background values.

## 3. Discussion

### 3.1. Expression Stability in Pear Leaves and Fruits

The level of transgene expression is affected by many factors related both to the plant and the environment [30,31,32]. Quantitative analysis showed a stable tendency of *uidA* gene expression in leaves of 19 transgenic pear lines during 12 years of field tests. Silencing of expression was not observed even during the extremely hot and dry season of 2010 [33] and after it. The variation of expression between pear lines with the intron-containing *uidA* gene could reach 130 times (from 0.12 to 13.7 pmol 4-MU/min/µg protein in 2010). This is much less than that reported by Cervera et al. [34] for citrange plants with the same gene under screenhouse conditions (from 0.1 to 154 pmol 4-MU/min/µg protein). It is generally assumed that expression variations between transgenic lines containing the same gene cassettes are caused by the position effect or epigenetic modification of the transgene [35]. Despite significant fluctuations in expression over the years, the relative differences between the lines with high and low expression were preserved. Li et al. [36] previously reported that herbicide resistance in *Populus* hybrids with the *bar* gene remained stable within three classes (tolerant, intermediate, and sensitive plants) for 8 years.

In fruits, the peel and pulp are studied more often (usually separately); seeds are analyzed less often. Since it is known that intensive histochemical GUS staining of vascular tissue may reflect not the level of expression but the penetration of the X-Gluc substrate [29], we included fruit peduncle in the analysis. Fluorimetric assay of *uidA* gene expression in different anatomical parts of pear fruit confirmed the pattern of histochemical staining. Expression in the pulp was many times lower than in other parts of the fruit—peel, peduncle, and seeds. The maximum expression was observed in seeds, slightly lower, in the peel and peduncle. Differences in the expression of transgenes in fruit tissues have been reported earlier only by Borejsza-Wysocka et al. [30]. The attacin E protein was highly expressed in the peel of apple fruits, but its content was low in unripe fruit pulp, and it was absent in ripe fruit pulp. This pattern of expression did not depend on the use of the PIN2 wound inducible promoter or the CaMV 35S constitutive promoter. In contrast to that work, in our study, the *uidA* gene under the control of the promoter CaMV 35S was expressed in the pulp of ripe pear fruits, though at a low level.

On the whole, the distribution of *uidA* gene activity in pear fruits is consistent with the distribution of biologically active compounds (phenolics and flavonoids), which the peel usually contains significantly more than the pulp of pear [37,38]. The content of arbutin, the characteristic compound of pear, was also 3–24-fold higher in the peel than in the pulp [39,40,41]. The contents of phenolic compounds, macro- and microelements in the apple peel were significantly higher than those in the pulp [42,43]. The fruit pulp exceeded the peel only in terms of sugar content [38,41,42]. Less is known about the chemical composition of seeds. A similar content of phenolic compounds in the peel and seeds, but a three–four-fold excess over pulp has been reported for fruits of pear [41] and mandarin [44]. Probably, the similarity of the pattern of transgene expression with the content of phenolic substances in fruit tissues (in the pulp less than in the peel and seeds) reflects the overall metabolic activity in ripe fruits.

### 3.2. Intron-Mediated Enhancement in Pear Trees

Most experiments on IME of expression in plants have been performed with the *uidA* gene since GUS enzyme activity can be detected directly in tissues [45]. The transformation of plants with the *uidA* gene containing intron IV2 of the potato gene *ST-LS1* was first reported in 1990 [46]. Later, this gene has been used in the transformation of many species, including fruit trees [34,47,48], but the expression has not been compared with an intron-free construct. Despite significant fluctuations in expression in some lines, stable IME of *uidA* gene expression was observed in pear leaves in the field. The degree of enhancement varied, depending on the year, from 1.4 to 2.3 times, which is slightly lower than that observed in the same pear lines in the greenhouse—from 2.5 to 3.7 times [49]. Our results are consistent with studies on herbaceous species. The magnitude of IME is usually in the range of 2- to 10-fold [50]. It is typically larger in monocots than dicots; for example, the addition of an intron of the *Arabidopsis thaliana UBI10* gene increased the expression of the alfalfa *NSP2* gene in barley by about 2.5 times [51].

IME of *uidA* gene expression was also observed in pear fruits. It was almost absent in the pulp (with the exception of the year 2007) but was similar between the peel, peduncle and seeds. The level of IME in these parts of the fruit was, on average twofold, which is comparable to the IME in leaves. The increased level of expression of the *uidA* gene with an intron also persisted during vegetative [52] and seed [53] propagation of transgenic pear plants.

### 3.3. Expression of Transgenes in Retransformants of Pear

The retransformed pear plants also showed stable expression without signs of silencing. Separation into lines with high and low expression was observed among lines of the same origin, although for retransformation, we used vegetative material. Several retransformed trees are known, but the stability of the first transgene expression was evaluated only on citrange with *uidA*-intron and *AP1* genes, which was retransformed with the *gfp* gene [54]. In contrast to our work, the level of AP1 transcripts in the retransformants varied within ±20% as compared with the baseline, despite the use of generative material for retransformation.

The long-term stability of herbicide resistance is difficult to assess on trees due to a significant increase in their aboveground part with age. Li et al. [36] coppiced *Populus* hybrids with the *bar* gene to evaluate herbicide resistance over 8 years. We treated individual branches of pear trees with herbicide in 2010, nine years after planting in the field. Almost all pear lines with the *bar* gene, including retransformed lines, showed their high resistance to herbicide, in the same way as earlier in 2002 and 2005 [49]. Signs of necrosis were observed only on the T-CF line, which was significantly less resistant to phosphinothricin (PPT) in vitro and to herbicide treatment in the greenhouse [55]. Thus, stability was present in the lines with both high and low resistance to the herbicide. The levels of resistance to the herbicide glufosinate remained stable in two transgenic *Populus* hybrids with *bar* gene in the field over 8 years [36]. However, unstable expression of the *bar* gene has been for transgenic *Populus alba* during cultivation in the greenhouse for two years [56]. 

### 3.4. Potential of Unintended Effects in Phenotype of Transgenic Trees

The introduction of even metabolically neutral genes, including the *uidA* gene, can cause unintended effects, for example, due to somaclonal variation or interactions at the site of insertion (position effect) [57]. Spatial positioning plays an important role in gene expression, and a number of organisms are known to cluster biosynthetic pathways for proper coregulation of expression. The disruption or alteration of gene expression due to the integration site of the transgene has been shown in plants [58] and fungi [59]. In particular, changes in the leaf proteome were observed in poplar with the *uidA* gene in vitro [60]. Transgenic *Populus deltoides* plants containing empty vectors unexpectedly demonstrated modified bud set, bud flush, and growth parameters after transfer from the greenhouse to the field [3]. Dwarf phenotypes were identified among hybrid poplar lines with the *TaLEA* gene [61] and birch lines with the *GS1* gene [62]. It is more difficult to assess unintended changes in plant physiology. In the boreal climate, at least a two-year trial is required to evaluate the growth of transgenic perennial plants [5]. Our two-year open-air tests revealed reduced frost tolerance in the aspen line with the *bar* gene [63].

In our study, qualitative phenotypic traits of transgenic pear trees have not changed. In addition, as an indicator of unintended changes, we evaluated the shape and size of the leaves. Leaf shape is considered a main factor that determines the plant structure and strongly influences plant performance [64]. Changes in leaf shape and size were observed in plums with one of three class 1 *KNOX* genes [65] and in poplar with the xyloglucanase *AaXEG2* gene [66]. High-throughput phenotyping tools based on computerized image analysis are more objective and accurate than traditional phenotyping methods and can be used for the evaluation of transgenic plants [67]. A quantitative imaging approach was used to evaluate differences in leaf morphology in transgenic Arabidopsis plants [68,69]. The shape and size of leaves of single- and double-transformed pear plants were determined using LAMINA software. Earlier, it has been used to detect significant changes in the shape of aspen leaves with the xyloglucanase gene [70]. A three-year study found no stable deviations in the shape and size of transgenic pear leaves. The existing changes in some lines were inconsistent and were not repeated in subsequent years. A high-throughput phenotyping analysis detects no consistent changes in transgenic pear fruit morphology, except for an increase in fruit weight and linear dimensions in one line [71]. This unintended effect may be beneficial since fruit size is an important agronomic trait.

### 3.5. Movement of Protein through Graft Union

There are many hypotheses about the mechanisms of long-distance signaling and rootstock–scion interactions [26]. Studies usually pay more attention to RNA transfer across grafts, but the results remain contradictory. On the one hand, the long-distance transfer over the graft union has been shown of siRNA in sweet cherry [23] and mRNA in pear [72]. On the other hand, there was no graft transmission of transgene mRNA in the grafted apple plants [73,74]. Little is known about transportable transgenic proteins in woody plants [26]. Nagel et al. [75] found no mRNA and GAFP-1 protein in the leaves of a wild-type scion on transgenic plum rootstock. pPGIP protein from transgenic grape rootstocks has been detected in the leaves of a non-transgenic scion but without any reverse transport [21]. Finally, the cry1Ac protein moved from the rootstocks to scions of triploid hybrid poplar and vice versa [76]. Most likely, the transport of a protein depends on its type, level of expression, distance from the grafting, and anatomical features of vascular tissue. Reports are known when the transport of transgenic protein, not the mRNA, through the graft, is observed [76,77].

Since it is known that the scion can also affect the rootstock [78,79], we analyzed the possible movement in both directions. β-glucuronidase activity was stable in the transgenic parts of the grafted plants for 10 years in the greenhouse and in the field, but no transfer through grafting in any direction was detected. Kodama et al. [27] did not detect mRNA of the *uidA* gene in the transcriptome of tomato grafted onto transgenic rootstock. Research into transport is usually carried out under controlled conditions. Nagel et al. [75] have not detected transgenic protein transport in plum plants in the greenhouse but suggest that it could accumulate in a non-transgenic scion after several years of growth in the field. Nevertheless, we found no transgenic protein after a prolonged growth of pear in the field. On the other hand, after 8 years of field growth, Bt protein was detected in all organs and tissues of a non-transgenic poplar scion or rootstock [80]. Thus, the issue of transgenic products’ transport through the graft requires further study. For example, the accelerated flowering of the scions grafted onto rootstocks expressing *FT* genes has been repeatedly demonstrated [24,81]. We have found early flowering in a birch line with the *GS1* gene, but its mechanism is not yet known [82]. This line can be used as a rootstock to study the transfer of the flowering acceleration signals to the scion.

## 4. Materials and Methods

### 4.1. Plant Materials

Transgenic pear plants with the *uidA* genes were obtained by *Agrobacterium*-mediated transformation of clonal rootstock GP 217 (*Pyrus communis* L.) according to Lebedev and Dolgov [83] in 1996–1997. These plants were transformed with the binary plasmid pBI121, containing 35S-*uidA* and nos-*nptII* genes (line name Nx), or the binary plasmid p35SGUSintron, containing 35S-*uidA* gene with the IV2 intron of the potato ST-LS1 gene [84] and nos-*hpt* gene (line name Hx). Plants were planted in the field (53° N, 36° E) in accordance with the permission of the Russian Inter-Agency Committee on Genetic Engineering Activity (authorization # 48-P/00) in 2000 [47]. Transgenic pear plants with herbicide resistance genes were transformed with the pBIBar plasmid containing the 35S-*bar* and the nos-*nptII* genes in 1999. For transformation, both wild-type plants (line name Px) and plants transformed with the pBI121 plasmid (line name Tx, T2x) were used [85]. The plants were planted in the field in 2001 (authorization # 31-P/00) [49]. The trees were managed according to standard practice for pear fruit production, including pruning, fertilization, irrigation, and pesticide treatment. Fruits were obtained by hand pollination of flowers from control and transgenic trees using mixed pollen of commercial pear cultivars [53] and harvested at the maturity stage (mid-August).

### 4.2. β-Glucuronidase (GUS) Assays

The expression of the *uidA* gene in flowers and fruits was evaluated using histochemical GUS staining. Flowers and fruits were cut, and longitudinal sections were incubated overnight in a X-Gluc solution at 37 °C [86]. The quantitative measurement of GUS activity in leaves and fruits was performed as described by Scott et al. [86] using 4-methyl-umbelliferyl-β-D-glucuronide (MUG) as substrate and the Tecan Infinite 200 microplate reader (Tecan Group Ltd., Männedorf, Switzerland) at Pushchino Center for Collective Use of Science Equipment. A mixture of leaves from the middle part of 3–5 branches of one tree (replication) was collected in early July. Mature fruits were separated into four parts: the peel, pulp, peduncle, and seeds (10–12 fruits from one tree as one replication). Leaves and fruit parts were grounded in liquid nitrogen, and powders were kept at −80 °C until extract preparation. Three biological replicates (trees) per genotype were used.

### 4.3. Tree and Leaf Phenotypic Assessment

Qualitative traits of whole trees and individual plant organs were visually assessed according to UPOV guidelines for pear [87] in the 2011–2013 field seasons. The assessment was conducted by comparison of control and transgenic trees with example cultivars using scale rate.

Leaves for high-throughput phenotyping were collected from control and transgenic lines in the 2011–2013 field seasons. A total of 20 middle-aged leaves per replication (tree) and three replications per genotype were used. Leaves were scanned using an HP Scanjet scanner at a resolution of 400 dpi, and images were saved as jpeg files for subsequent analysis. The size and shape parameters of leaves were quantified with LAMINA 1.0.2 (Leaf shApe deterMINAtion) software [88]. Leaf length was defined manually as the distance between the leaf apex and the base of the petiole. The eight leaf parameters—area, perimeter, circularity, length, width, length:width, horizontal symmetry, and vertical symmetry—were measured using LAMINA 1.0.2 software.

### 4.4. Herbicide Application

Seventeen transgenic pear lines expressing the *bar* gene and untransformed control were evaluated for herbicide resistance in 2010. Three trees were used for each line. Three to four branches from each tree were treated with a 1% aqueous solution of the herbicide Basta (Bayer CropScience, Leverkusen, Germany, 150 g/L PPT) using a backpack sprayer. The application rate of herbicide was respectively 10 L/ha (double field dose). Leaf damage was scored 3 and 7 days after application.

### 4.5. Grafting Experiments

Greenhouse-grown potted pear plants were used for grafting experiments. Non-transgenic and transgenic plants expressing the intron-free (line NII-1) and intron-containing (line HB-4) *uidA* genes were micropropagated and cultivated for one year in the greenhouse. In the spring of 2000, two buds from the control and transgenic plants were grafted onto the transgenic and control plants, respectively. Two grafting combinations were constructed: (1) WT tip and lateral shoot grafted onto a transgenic rootstock with one lateral shoot, (2) transgenic tip and lateral shoot grafted onto a non-transgenic rootstock with two lateral shoots (Figure 7). In total, five plants were obtained for each combination: two with line HB-4 and three with line NII-1. Grafted plants were transplanted in the field in 2007. GUS activity was quantified in leaves of tip and lateral shoots under greenhouse (2000 and 2001) and field (2008 and 2009) conditions as previously described. 

### 4.6. Statistical Analysis

Data are presented as mean ± standard error (SE). All data were tested by ANOVA using Statistica 10 software (StatSoft Inc., Tulsa, OK, USA). Means were separated by the Duncan test at a significant level of 0.05.

## 5. Conclusions

We showed the stability of *uidA* gene expression in field-grown pear trees over 16 years after transformation (1997–2013). The oldest transgenic trees described in the literature are hybrid aspen lines that have confirmed the stability of the *rolC* gene expression in glasshouse-grown trees up to 18 years [2]. There was no silencing either in the pear leaves or fruits or in single- and double-transformed pear plants. The absolute level of expression varied significantly over the years, but the relative differences between the lines (low, intermediate and high expression levels) remained. Plants containing the gene with an intron had a higher expression, thus confirming the stable IME in fruit trees in the field. Herbicide resistance in plants with the *bar* gene was also stable over a long time. Experiments with grafted pear plants showed that the GUS protein was not transported through grafting in any direction. These results may be useful for enhancing expression, gene pyramiding, and assessing the biosafety of transgenic trees.

## Figures and Tables

**Table 1:** Fluorimetric estimation of GUS activity in pear leaves.

Transgene	Line	4-MU, pmol/min/µg Protein							
		2001	2003	2005	2006	2007	2009	2010	2011
control	GP217	1	1	1	1	1	1	1	1
*uidA*-intron	HA-1	59	45	197	156	220	429	118	211
	HA-2	101	24	36	29	6	57	14	17
	HA-3	66	84	184	89	164	387	716	194
	HA-4	113	83	342	131	314	209	267	216
	HA-5	59	55	211	141	132	367	95	226
	HA-6	210	23	323	88	35	92	14	56
	HA-7	28	10	23	11	5	8	6	5
	HB-1	242	214	675	105	100	786	242	393
	HB-2	157	30	560	256	133	199	103	72
	HB-4	249	51	465	143	28	114	17	31
*uidA*	NI-1	10	6	37	3	3	10	16	3
	NII-1	44	66	213	163	234	349	87	266
	NII-2	40	27	58	68	95	135	45	107
	NII-3	238	60	443	108	53	240	106	195
	NII-4	72	8	159	35	98	350	64	152
	NIII-2	66	24	79	82	70	45	63	67
	NIII-4	86	62	57	114	61	181	170	55
	NIII-5	22	21	113	7	24	9	42	26
	NIV-2	96	20	73	35	29	141	35	13

Data are represented as a normalized relative value of a non-transgenic line. The color gradient represents the change in GUS activity from the minimum (white) to the maximum (red) value.

**Table 2:** Expression of the *uidA* gene in the leaves of pear retransformants.

Line	Initial	4-MU, pmol/min/µg Protein							
	Line	2002	2003	2005	2006	2007	2009	2010	2011
GP217	-	1	1	1	1	1	1	1	1
P-BK	GP217	2	1	0	1	0	1	1	1
T-AP	NII-1	92	50	591	21	185	353	147	467
T-BO	NII-1	8	4	21	3	8	7	5	15
T-BT	NII-1	36	24	152	18	236	82	288	242
T-CD	NI-1	3	3	5	4	6	3	2	15
T-CF	NII-1	6	7	112	6	49	6	24	30
T2-AF	NIII-2	5	4	11	3	8	12	9	20
T2-BF	NIII-4	45	22	82	19	118	121	123	242
T2-BR	NIV-2	33	39	109	36	82	129	64	287
T2-BS	NIV-2	78	21	204	12	47	91	62	313
T2-DE	NIV-2	6	3	7	4	5	7	5	19
T2-ES	NIII-4	91	40	131	41	114	172	173	312

Data are represented as a normalized relative value of a non-transgenic line. The color gradient represents the change in GUS activity from the minimum (white) to the maximum (red) value.

**Table 3:** Expression of *uidA* gene in leaves of transgrafted pear (pmol 4-MU/min/µg protein).

Line	Greenhouse		Field	
	2000	2001	2008	2009
GP217	0.09 ± 0.01	0.03 ± 0.00	0.03 ± 0.00	0.03 ± 0.00
HB-4	10.6 ± 1.5	5.5 ± 0.7	5.8 ± 1.9	5.6 ± 1.7
NII-1	3.0 ± 0.5	8.0 ± 1.1	5.9 ± 1.8	8.2 ± 1.2

Data are means (for both grafting schemes) ± SE (*n* = 20 for GP217; *n* = 6 for HB-4; *n* = 8 for NII-1).

## Data Availability

Not applicable.

## Supplementary Materials

The following supporting information can be downloaded at: https://www.mdpi.com/article/10.3390/ijms241612883/s1.
