# Supplementary material for: Impact of Intron and Retransformation on Transgene Expression in Leaf and Fruit Tissues of Field-Grown Pear Trees
Source: Int J Mol Sci. 2023 Aug 17;24(16):12883. doi: 10.3390/ijms241612883 (PMC10454629; doi:10.3390/ijms241612883)
Supplement: Supplementary file 1 [file ijms-24-12883-s001.zip › ijms-2502457-supplementary.pdf]

Supplementary Materials

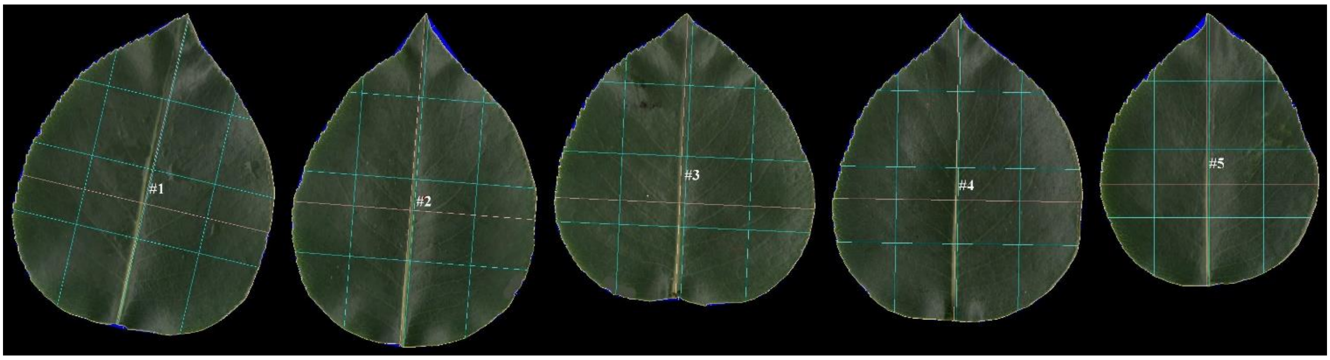

Figure S1. Pear leaf images after processing by LAMINA software.

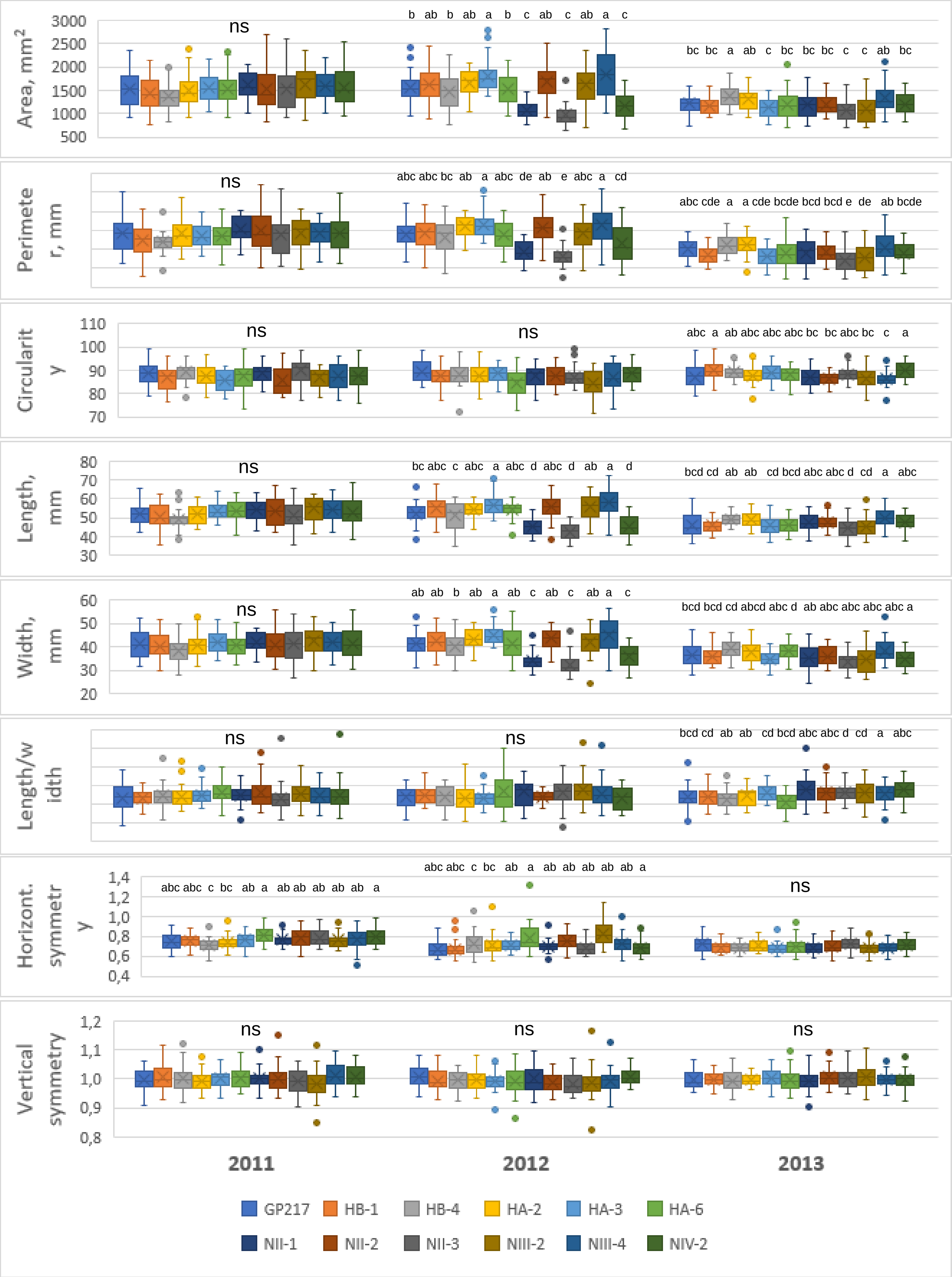

Figure S2. Leaf size and shape values for transgenic pear plants with *uidA* gene. Boxplots shown with median (horizontal line), cross for mean, box for 2nd and 3rd quartiles and whisker for maximum and minimum values (n=60). Outliers are shown as individual circles. Different letters indicate significant differences at P<0.05, ns = not significant.

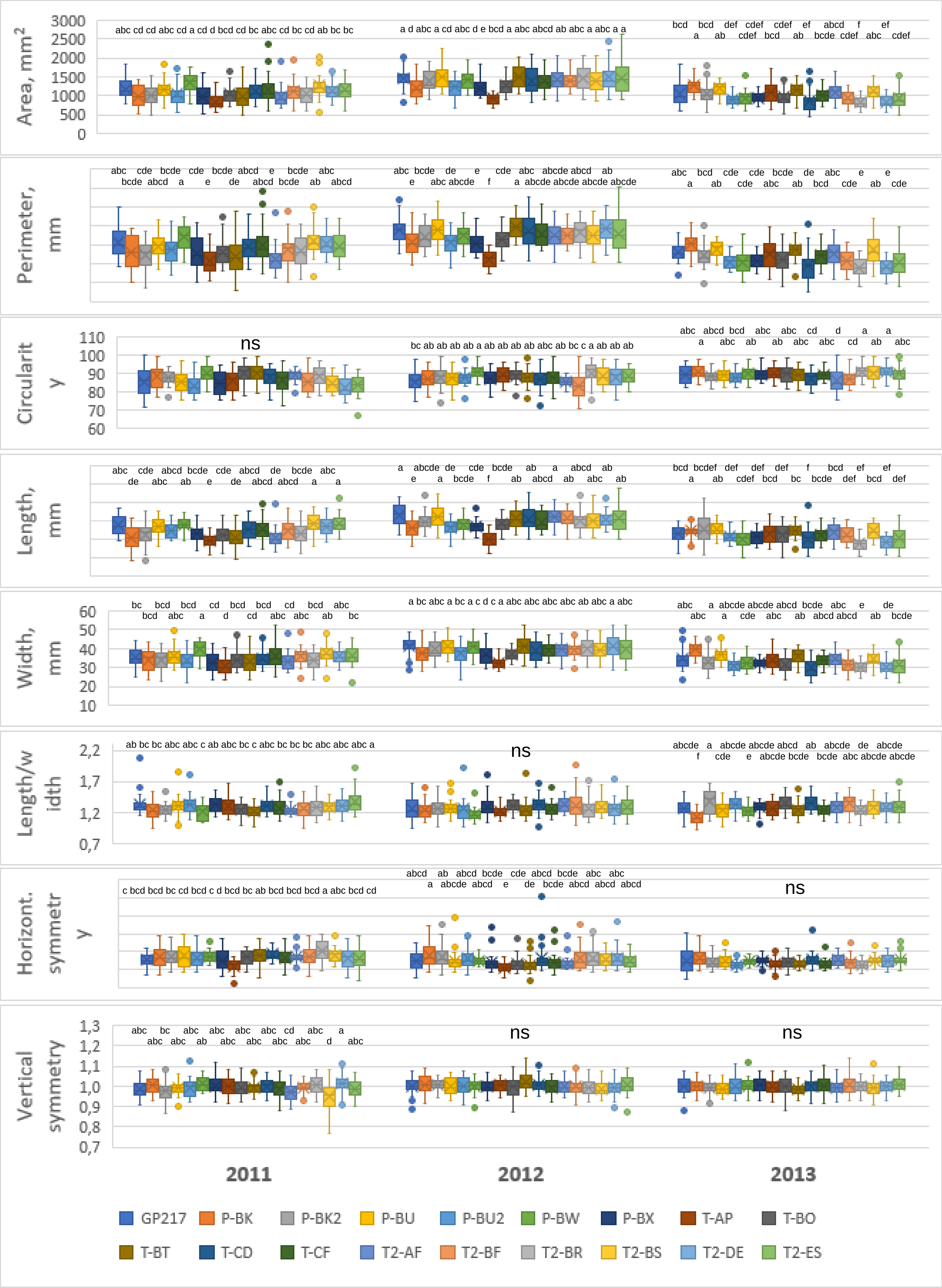

Figure S3. Leaf size and shape values for transgenic pear plants with *bar* gene. Boxplots shown with median (horizontal line), cross for mean, box for 2nd and 3rd quartiles and whisker for maximum and

minimum values (n=60). Outliers are shown as individual circles. Different letters indicate significant differences at P<0.05, ns = not significant.

Table S1. Expression of *uidA* gene in parts of pear fruits.

Line	Part of fruit	4-MU, pmol/min/µg protein				
		2007	2009	2010	2011	2013
GP217	peel	0.04±0.01	0.02±0.01	0.04±0.02	0.03±0.01	0.05±0.01
	pulp	0.01±0.00	0.01±0.00	0.01±0.00	0.01±0.00	0.01±0.00
	peduncle	0.03±0.01	0.03±0.01	0.06±0.01	0.04±0.01	0.04±0.00
	seeds	0.01±0.00	0.02±0.01	0.01±0.00	0.01±0.00	0.02±0.01
HA-2	peel	2.0±0.6	0.3±0.1	0.7±0.1	4.5±1.1	3.7±0.8
	pulp	0.4±0.1	0.1±0.0	0.1±0.0	0.3±0.1	0.2±0.0
	peduncle	2.1±0.4	0.5±0.1	0.6±0.1	1.5±0.5	0.9±0.2
	seeds	5.1±1.0	6.1±0.5	6.3±1.0	10.7±2.7	2.8±0.7
HA-3	peel					5.7±0.9
	pulp					0.5±0.1
	peduncle					4.0±0.8
	seeds					9.7±0.8
HA-6	peel		1.0±0.1	0.9±0.3	5.4±1.3	3.8±0.5
	pulp		0.1±0.0	0.1±0.0	0.2±0.0	0.1±0.0
	peduncle		2.3±0.6	1.6±0.2	5.0±1.2	1.9±0.4
	seeds		2.0±0.3	4.1±0.1	3.4±0.5	2.1±0.5
HB-1	peel	6.0±1.7	1.4±0.3	1.8±0.5	5.1±1.3	3.6±0.8
	pulp	0.8±0.2	0.1±0.0	0.2±0.0	0.3±0.0	0.1±0.0
	peduncle	8.3±2.3	4.0±0.9	6.3±0.6	6.7±1.4	4.4±1.0
	seeds	2.0±0.4	4.9±0.6	3.5±0.8	11.9±2.2	4.4±0.8
HB-4	peel	3.9±0.7				5.1±0.7
	pulp	0.7±0.2				0.4±0.1
	peduncle	1.5±0.4				2.8±0.4
	seeds	8.1±1.4				16.2±2.2
NII-1	peel	1.1±0.3	0.2±0.0	0.9±0.3	5.0±0.9	5.8±0.9
	pulp	0.2±0.1	0.1±0.0	0.1±0.0	0.5±0.1	0.2±0.0
	peduncle	0.8±0.2	0.6±0.2	2.8±0.6	2.6±0.7	1.4±0.3
	seeds	4.6±0.8	2.3±0.4	4.1±0.6	4.8±0.7	2.6±0.7
NII-2	peel	0.5±0.2	0.7±0.2	1.1±0.2	1.9±0.3	3.6±0.4
	pulp	0.2±0.0	0.2±0.0	0.1±0.0	0.2±0.1	0.4±0.1
	peduncle	2.6±0.3	2.2±0.3	3.4±0.7	3.8±0.7	1.8±0.6
	seeds	1.0±0.2	0.7±0.2	3.3±0.7	3.1±0.6	0.8±0.2
NII-3	peel		0.4±0.1	0.2±0.1	1.3±0.2	2.9±0.7
	pulp		0.1±0.0	0.1±0.0	0.1±0.0	0.3±0.1
	peduncle		0.7±0.1	0.3±0.1	1.6±0.4	3.3±0.8
	seeds		3.1±0.7	2.0±0.5	3.0±0.3	5.4±1.4
NIII-2	peel	1.1±0.3	0.5±0.1	1.7±0.3	2.3±0.6	2.3±0.5
	pulp	0.2±0.0	0.1±0.0	0.5±0.1	0.2±0.0	0.1±0.0
	peduncle	2.2±0.3	1.7±0.5	5.1±0.8	2.9±0.6	1.3±0.3
	seeds	0.7±0.2	0.8±0.2	3.6±0.6	2.9±0.9	2.3±0.6
NIII-4	peel		0.2±0.0	1.0±0.2	4.7±1.2	2.3±0.5

	pulp<br>peduncle<br>seeds		0.1±0.0<br>0.5±0.2<br>2.2±0.1	0.2±0.1<br>0.9±0.2<br>2.5±0.5	0.3±0.1<br>1.9±0.4<br>2.9±0.7	0.2±0.0<br>2.1±0.3<br>3.2±0.6
NIV-2	peel<br>pulp<br>peduncle<br>seeds		0.3±0.1<br>0.1±0.0<br>0.3±0.1<br>0.9±0.2	0.5±0.1<br>0.2±0.0<br>1.1±0.2<br>2.2±0.4	2.3±0.5<br>0.2±0.0<br>1.9±0.5<br>2.6±0.5	1.7±0.4<br>0.1±0.0<br>1.2±0.3<br>2.7±0.7

Data are means ± SE (n=3).

Table S2. Phenotypic traits for assessment of control and transgenic pear trees.

Plant organ	UPOV trait No.	Trait	Characteristics	UPOV scale No.
Tree	1	Vigor	between weak and medium	4
	2	Branching	strong	7
	3	Habit	spreading	4
One-year-old shoot	4	Growth	wavy	2
	5	Length of internode	medium	5
	6	Predominant color on sunny side	brown red	5
	7	Number of lenticels	medium	5
	8	Shape of apex of vegetative bud	acute	1
	9	Position of vegetative bud in relation to shoot	slightly held out	2
	10	Size of bud support	large	7
Young shoot	11	Anthocyanin coloration of growing tip (during rapid growth)	absent or very week	1
	12	Intensity of pubescence (upper third)	absent	1
Leaf blade	13	Attitude in relation to shoot	outwards	2
	17	Shape of base	right-angled	2
	18	Shape of apex (excluding pointed tip)	right-angled	2
	19	Length of pointed tip	short	3
	20	Incisions of margin (upper half)	sharply serrate	4
	21	Depth of incisions of margin	shallow	3
	22	Curvature of longitudinal axis	weak	3
Petiole	24	Presence of stipules	present	9
	25	Distance of stipules from basal attachment of petiole	short	3
Flower	29	Attitude of sepals in relation to corolla	recurved	3
	30	Position of margins of petals	touching	2
	31	Position of stigma in relation to stamens	same level	2
	33	Shape of petal (excluding the claw)	curcular	1
Immature fruit	34	Color of sepals (early summer)	red-brown	4
Fruit	43	Profile of sides	straiht	2
	45	Relative area of over color	absent or very small	1
	52	Curvature of stalk	absent or very small	1

	53	Attitude of stalk in relation to axis of fruit	oblique	2
	55	Attitude of sepals (at harvest)	spreading	3
	59	Relief of area around eye (at harvest)	smooth	1
Seed	63	Shape	ovate	2
